# Biological effects of *Lippia alba* essential oil against *Anopheles gambiae* and *Aedes aegypti*

**DOI:** 10.1038/s41598-024-52801-1

**Published:** 2024-02-12

**Authors:** Fangala Hamidou Coulibaly, Marie Rossignol, Mohamed Haddad, David Carrasco, Alain Azokou, Adeline Valente, Carole Ginibre, Mamidou Witabouna Koné, Fabrice Chandre

**Affiliations:** 1 UMR MIVEGEC (Montpellier University/IRD/CNRS), 911 avenue Agropolis, 34394 Montpellier Cedex 5, France; 2 Université Nangui Abrogoua, 02 BP 801, Abidjan, Côte d’Ivoire; 3 Centre Suisse de Recherches Scientifiques en Côte d’Ivoire, 01 BP 1303, Abidjan 01, Côte d’Ivoire; 4 UMR 152 Pharma Dev, Université de Toulouse, IRD, UPS, 35 chemin des Maraîchers, 31062 Toulouse Cedex 9, France

**Keywords:** Animal behaviour, Entomology, Disease prevention, Infectious diseases

## Abstract

The management of mosquito resistance to chemical insecticides and the biting behaviour of some species are motivating the search for complementary and/or alternative control methods. The use of plants is increasingly considered as a sustainable biological solution for vector control. The aim of this study was to evaluate the biological effects of the essential oil (EO) of *Lippia alba* harvested in Abidjan (Côte d’Ivoire) against *Anopheles gambiae* and *Aedes aegypti* mosquitoes. Phytochemical compounds were identified by GC–MS. Knockdown and mortality were determined according to the WHO test tube protocol. Contact irritancy was assessed by observing the movement of mosquitoes from a treated WHO tube to a second untreated tube. Non-contact repellency was assessed using a standardised high-throughput screening system (HITSS). Blood meal inhibition was assessed using a membrane feeding assay treated with EO. The EO was identified as the citral chemotype. The EO gave 100% KD60 in both species at a concentration of 1%. Mortalities of 100% were recorded with *An. gambiae* and *Ae. aegypti* at concentrations of 1% and 5% respectively. The highest proportions of females escaping during the contact irritancy test were 100% for *An. gambiae* at 1% concentration and 94% for *Ae*. *aegypti* at 2.5% concentration. The 1% concentration produced the highest proportions of repelled mosquitoes in the non-contact repellency tests: 76.8% (*An. gambiae*) and 68.5% (*Ae. aegypti*). The blood meal inhibition rate at a dose of 10% was 98.4% in *Ae*. *aegypti* but only 15.5% in *An. gambiae*. The citral chemotype of *L. alba* EO has promising biological effects in both species that make it a potentially good candidate for its use in mosquito control. The results obtained in this study encourage the further evaluation of *L. alba* EOs from other localities and of different chemotypes, under laboratory and field conditions.

## Introduction

Malaria, the most widespread parasitic disease in the world, is the deadliest of the diseases whose parasites are transmitted by mosquitoes^1^. The morbidity of arboviral diseases such as dengue, chikungunya, yellow fever and Zika virus fever is also a major concern. For example, despite the availability of a vaccine, yellow fever affects 130,000 people and causes 500 deaths each year in the WHO African region alone, where more than 440 million people are at risk^2^. Control of *Anopheles* and *Aedes* mosquitoes, vectors of malaria pathogens and arboviruses respectively, is therefore essential to prevent these vector-borne diseases.

The main methods of vector control adopted by national control programmes and promoted by the WHO are mainly based on chemicals^1–3^. For instance, as part of the fight against malaria vectors, 459 million long-lasting insecticidal nets (LLINs) were distributed worldwide between 2015 and 2017. As for the control of mosquitoes that transmit arboviruses, national programmes use indoor residual spraying (IRS) and spatial spraying of insecticides^4,5^. Unfortunately, the effectiveness of these tools is getting limited due to the evolution of resistances in most of mosquito vector species to the insecticide molecules recommended for public health use^2,4,6–8^. These resistances are presumed to be due to the use of insecticides in domestic hygiene and public health, and more importantly to the use of the same insecticides types in agricultural production areas^9^. According to Agossa et al.^10^, the effectiveness of LLINs decreases considerably in areas of high vector resistance. In addition to the ability of mosquito populations to develop resistance mechanisms, these chemicals can persist in the environment and poison non-target organisms^11^. Chemical control also changes the behaviour of vectors^12^. Indoor residual spraying and LLINs are thought to be at the base of the observed switch in *Anopheles* mosquitoes biting patterns, *i.e.* from biting majoritarily indoors towards preferentially biting outdoors when human hosts are not protected^13–15^, which may explains the persistence of high residual transmission of malaria in some areas^15–17^. These concerns associated with chemical control are driving research into new and more environmentally friendly vector control alternatives. In this context, several studies have been carried out on the biological activities of natural plant extracts against vector mosquito species^18–24^.

Several studies present plant extracts as potential alternatives for mosquito control. For instance, Dua et al.^25^ showed that a chloroform fraction of fresh *Lantana camara* (Verbenaceae) flowers prevent 100% of *Aedes albopictus* bites for 3 h 45 min in the laboratory and 76% for 7 h in the field against mosquitoes of the genus *Aedes*. Carotol, a major component of the essential oil (hereafter EO) extracted from carrot seeds (*Daucus carota sativus* L., Apiaceae), has also shown good repellent activity against *Aedes aegypti* and *Anopheles quadrimaculatus*^26^. *Artemisia argyi* (Asteraceae) EOs collected from seven sites in China have all shown significant repellent activity on *Anopheles sinensis*^27^.

*Lippia alba* (Verbenaceae) is an aromatic medicinal plant native of South America. It is used by local people for its medicinal (e.g. antimalarial, antiviral, digestive, respiratory, sedative and antihypertensive) and culinary properties^28–31^. Studies have highlighted its antioxidant, antibacterial, anti-genotoxic, anti-inflammatory and neuro-sedative activities^32–37^. Unlike certain aromatic plants, *L. alba* has been understudied to evaluate its biological effects on mosquitoes. EOs extracted from leaves and stems harvested in Colombia showed adulticidal activity on *Ae. aegypti*^38^. The oil extracted from the whole plant harvested in Bucaramanga (Colombia) showed significant adulticidal activity against *Ae. aegypti*, in contrast to the EO from leaves harvested in India (Jalukbari, Guwahati, Assam) on adults of *Ae. aegypti* and *Culex quinquefasciatus*^39,40^. *L. alba* EO could be used as topical repellent^40–42^, although the one evaluated by Castillo et al.^39^ did not repel *Ae. aegypti* females. These results prompted the evaluation of this plant species grown in Côte d'Ivoire against *Anopheles gambiae* and *Ae. aegypti*, as the phytochemical composition of plants can vary according to their genetic diversity or environmental conditions. This paper presents the phytochemical composition of the EO of *L. alba* harvested in Abidjan (Côte d'Ivoire) and its adulticidal, repellent and blood meal inhibiting effects on *An. gambiae* and *Ae. aegypti*.

## Methods

### Cultivation of *L. alba* and extraction of the essential oil

Ornamental *L. alba* plants were harvested and planted in our experimental field at Nangui Abrogoua University (5° 23ʹ 19ʺ N, 4° 0ʹ 54ʺ W) in February 2021. The species was previously identified at the herbarim of the Centre Suisse de Recherches Scientifiques en Côte d’Ivoire with reference to specimen 009947. Cuttings of 12–17 cm were planted in nursery bags filled with organic substrate from a former poultry farm waste pit. The plants were then watered in the morning and in the evening during one month and then once a day until harvest in May 2021.

The EO was extracted from the leaves by hydro-distillation using a Clevenger^43–46^. Five hundred and thirty (530) grams of fresh leaves were placed over 2 L of water in a pressure cooker. 4.6 mL of EO were obtained after two hours of distillation. The EO was stored in an 8 mL shaded bottle at 4 °C.

### Mosquitoes

Bioassays were carried out on mosquitoes of the genus *Aedes* and *Anopheles* susceptible to chemical insecticides and maintained at the IRD insectarium (27 ± 1 °C and 70 ± 10% relative humidity) in Montpellier: Kisumu strain of *An*. *gambiae* from Kenya and SBE strain of *Ae*. *aegypti* from Benin. The photoperiod was set at 14 h light and 10 h dark.

### GC–MS data acquisition

GC–MS was used to identify the essential oil compounds. 1 µL of a 2% solution of the essential oil diluted in hexane was injected into the Agilent 8860 System chromatograph coupled to the Agilent 5977B GC/MSD mass spectrometer. The oven temperature was initially set at 50 °C for 2 min, before being increased to 110 °C (4 °C/min), 250 °C (8 °C/min) and 310 °C (10 °C/min). The carrier gas was helium at a temperature of 250 °C, a pressure of 7.6522 psi and a total flow rate of 104 mL/min in split mode. The Agilent 122-5032 column (30 m × 250 μm × 0.25 μm) had a constant flow rate of 1 mL/min. The temperature of the mass detector was 300 °C.

### Data preprocessing of the GC–MS data

GC–MS data were first analyzed and processed using MzMine 2.53 to visualize the chromatograms and spectra^47,48^. Then, GC–MS raw data were converted to Analysis Base File (ABF) format using ABF converter (http://www.reifycs.com/AbfConverter/index.html). Next, the converted data were pre-processed using MS-DIAL 4.70 (NSF-JST, Japan), including peak extraction, peak alignment, baseline calibration, deconvolution analysis and peak identification. We selected KovatsRI based on alkanes as a retention index for peak alignment. The deconvoluted spectra in NIST MSP format (GC–MS DB-Public-KovatsRI-VS3) were imported and matched with spectral libraries. Those peaks with an average peak width of 20 scans and minimum peak height above 10,000 amplitudes were selected for peak detection. Peaks with a s-window value of 0.5 and EI spectral cutoff of 5000 amplitudes underwent a deconvolution operation. The identification parameters were set as follows: m/z tolerance of 0.5 Da, retention time tolerance of 0.5 min, EI similarity cutoff value of 70% and identification score cutoff value of 70%. The alignment parameters of retention time tolerance and retention time factor were set to 0.075 min and 0.5, respectively.

### Adulticidal tests

The insecticidal activity of the EO of *L. alba* was evaluated at dilutions of 0.1%, 1%, 2.5% and 5% (v/v). Permethrin (91.8% technical grade) was used as a positive control (0.011 and 0.11 mg/cm^2^). The EO was diluted in a solution of ethanol plus silicone oil Dow Corning 566 (134 mL ethanol + 66 mL silicone) and permethrin in a solution of acetone plus silicone oil Dow Corning 566 (134 mL acetone + 66 mL silicone). Two milliliters of the different solutions were used to impregnate the filter papers (12 × 15 cm, Whatman®). The tests were conducted with slight modifications to the protocol of Deletre et al.^21^, at 27 ± 2 °C and 80 ± 10% relative humidity. Two WHO tubes were used, one marked with a red dot for mosquito exposure to impregnated paper and one marked with a green dot containing unimpregnated paper for mosquito holding. A total of 25–30 females aged 3–7 days (post-emergence) were introduced into three holding tubes. After 30 min of acclimatation, the dead mosquitoes, if any, were replaced. The mosquitoes were then transferred to the exposure tubes for 60 min. After exposure time, knocked down mosquitoes (KD60) were counted and all the mosquitoes were transferred back to the holding tubes. Tubes were kept in a climatic chamber (27 ± 2 °C and 80 ± 10% relative humidity) for 24 h. During this time, individuals were provided with a cotton soaked with a 10% honey solution for feeding. The number of dead mosquitoes were checked at 24 h after exposure. For each product and concentration, mosquitoes exposed to papers impregnated with the solvent only served as negative controls. Mortality for treated mosquitoes was corrected by the Abbott formula when mortality in control mosquitoes was between 5 and 20%: (%treatment mortality−%control mortality)/(100−control mortality) × 100.

### Contact irritancy tests

The contact irritancy of the EO was evaluated at concentrations of 0.1%, 1%, 2.5% and 5%. The tests were performed in WHO tubes. Two tubes (one marked with a red dot and the other with a green dot) were connected with a slide door unit and placed horizontally on the laboratory bench. Whatman® paper (12 × 15 cm) impregnated with the test solution (2 mL) was placed in the red-dotted tube and unimpregnated paper in the green-dotted tube. The papers were held against the walls of the tubes with metal clips. The device for the negative control consisted of a tube containing a solvent impregnated paper (ethanol and silicone for the essential oil, acetone and silicone for permethrin) and a tube containing unimpregnated paper. Twenty to twenty-four (20–24) females aged 3–7 days after adult emergence were introduced into the tube containing the impregnated paper. After 30 s of acclimatation, the slide unit door was opened and closed after 10 min. Mosquitoes were then killed in a freezer. Mosquitoes present in each tube were then counted and the proportion of passage from treated to untreated tube was determined^21,49^. Permethrin was evaluated as a positive control. The test was validated when the proportion of escaped mosquitoes in the negative controls was less than 50%. The test was performed three times for each dose. Tests were performed at 27 ± 2 °C and 80 ± 10% relative humidity.

### Non-contact repellency tests

**Figure 1:**
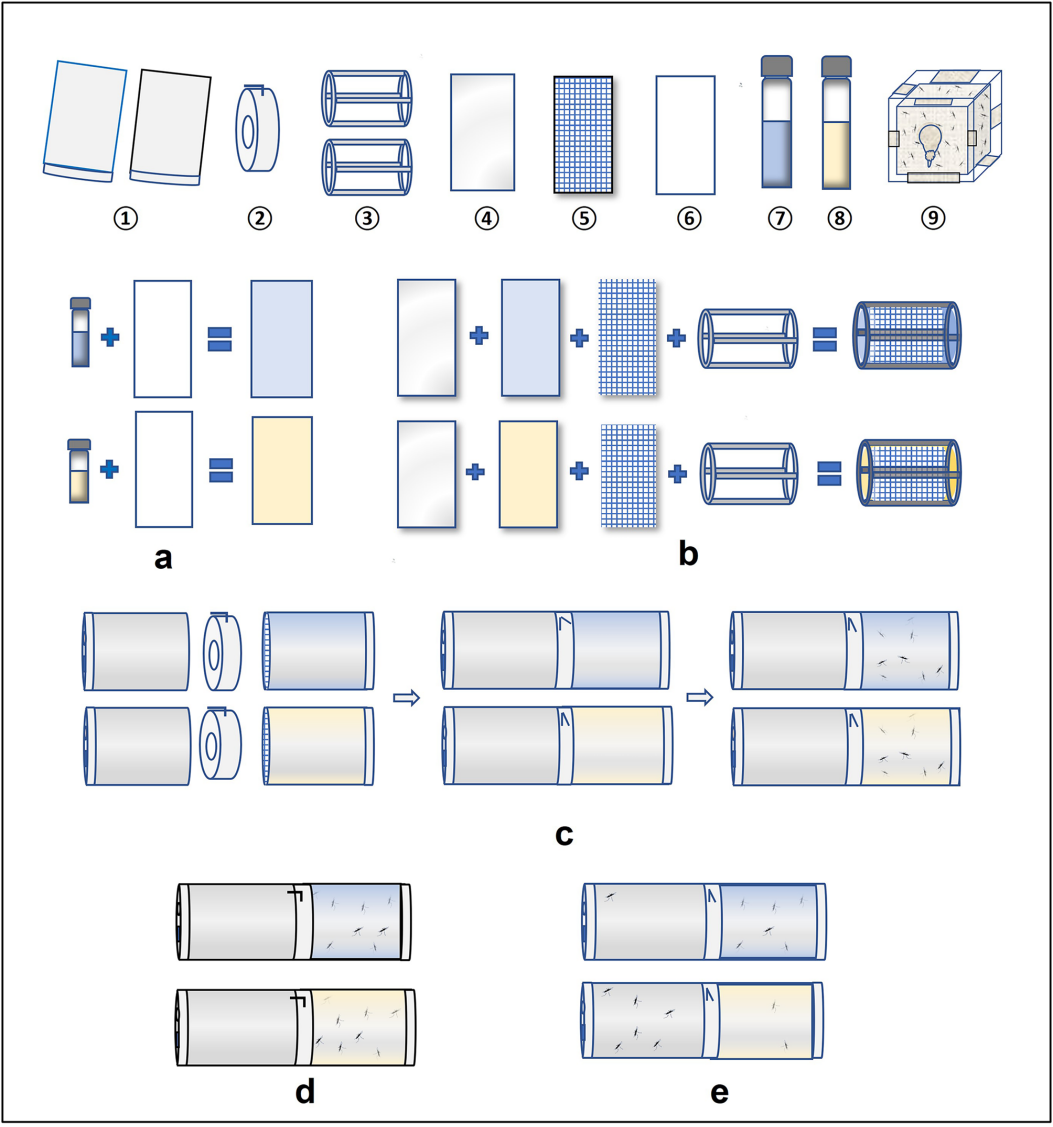
Steps to perform the non-contact repellency test. **①** : HITSS metal cylinders, **②** : Linking section, **③** : drum, **④** : Rigid transparent film, **⑤** : Insect screen, **⑥** : Whatman paper (30 × 10 cm), **⑦** : Control solution, **⑧** : EO solution, **⑨** : Mosquitoes. (**a**) Paper impregnation, (**b**) positioning paper around drum, (**c**) connecting the cylinders and introducing the mosquitoes into the treated cylinders, (**d**) opening of the link section doors after 30 s of acclimatation, (**e**) link section doors closed after 10 min and mosquitoes counted.

The repellent effect of the EO of *L. alba* was evaluated using a modified High-Throughput Screening System inspired by the methodology proposed in Grieco et al.^50^ and Deletre et al.^21^. The system consisted of two cylindrical chambers (treated and untreated) connected by a door. Whatman paper (10 × 30 cm) impregnated with the solution to be tested (3.3 mL) was placed between a rigid transparent film and a mosquito net. The assembly was rolled around a drum so that the transparent film was on the exterior of the armature and the whole was placed in the treated chamber. Unimpregnated paper was placed in the untreated chamber. The treated chamber for the control mosquitoes contained paper impregnated only with solvent (ethanol–silicone oil Dow Corning 566). Twenty to twenty-four fasting females aged 3–7 days (post-emergence) were introduced into the treated chamber. The door was opened after 30 s of acclimatation and closed again after 10 min (Fig. 1). Mosquitoes in each of the chambers were anesthetized with CO_2_ and then counted. The proportion of passage from the treated to the untreated chamber was then determined. The essential oil was evaluated at 0.1, 1, 2.5 and 5%. The test was performed three times for each dose and was validated when the proportion of escaped mosquitoes in the negative controls was less than 50%. Bioassays were carried out at 27 ± 2 °C and 80 ± 10% relative humidity.

### Blood feeding inhibition

**Figure 2:**
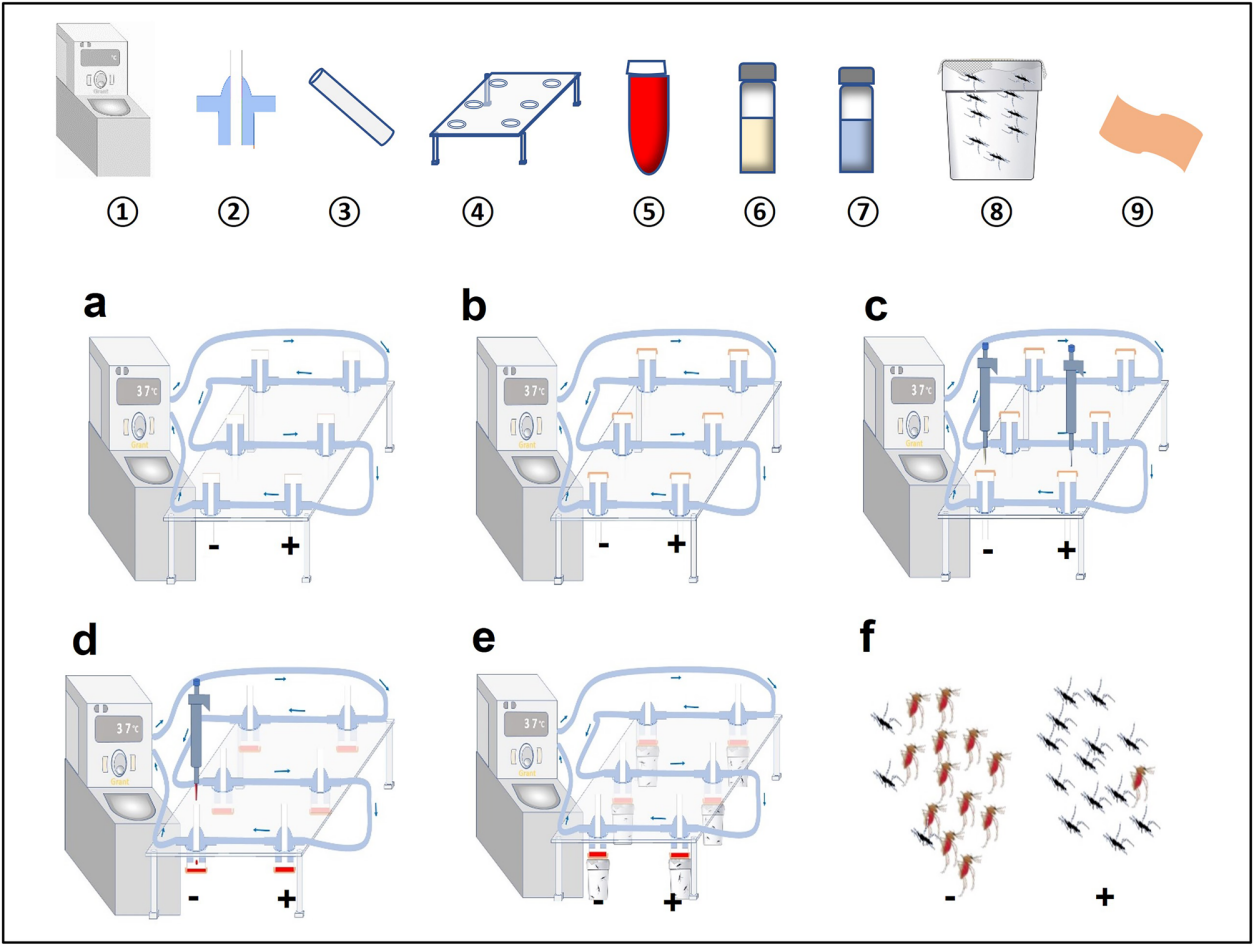
Descriptive diagram of the blood meal inhibition test. **①**: Water Bath, **②** : Feeder **③** : Pipe, **④** : Rack, **⑤** : Rabbit blood, **⑥** : Essential oil solution, **⑦** : Control solution, **⑧** : Mosquitoes, **⑨** : Pig membrane. **−**: Control line, + : treated line. (**a**) Setting up the system. (**b**) Laying of pig membranes on the bottom surfaces of feeders. (**c**) Impregnation of pig membranes. (**d**) Introduction of blood into feeders. (**e**) Place mosquito cups underneath the feeders for 1 h. (**f**) Count of blood fed and non fed mosquitoes.

Solutions of *L. alba* EO at 1%, 2.5%, 5% and 10% were prepared with absolute ethanol (v/v). The first three solutions were evaluated before the 10% essential oil solution and ethanol was used as a negative control for each test. Glass feeders (Ø = 16.32 mm) were connected to each other by silicon tubing: 12 feeders for the evaluation of the first 3 solutions or 6 feeders for the evaluation of the 10% solution. They were then connected to a water bath equipped with a pump to circulate water through the feeders at 37 °C. The feeders were positioned on the racks at a rate of three feeders per dose. Pre-cut pig gut membranes were placed on the underside of the feeders. Ten (10) µL of the EO solutions were spread on each membrane. The feeders were then turned upside down and 100 µL of rabbit blood was introduced into each feeder. Cups (440 mL) covered with mosquito netting containing 20–29 adult females aged 5–10 days old were placed under each feeder. After one hour, the mosquitoes were removed and stunned in the freezer before counting the blood fed mosquitoes (Fig. 2). The test was validated when the feeding rate in control mosquitoes was at least 75%. The blood meal inhibition rate was defined as: [1−(% fed EO/% fed Ethanol)] × 100. The tests were carried out in 3 replicates at 27 ± 2 °C and 80 ± 10% relative humidity.

### Statistical analysis

The data were analysed using R-4.2.1 software^51^. The proportions of KD60, dead or displaced mosquitoes induced by the essential oil were compared to those induced by the different negative controls using Fisher exact test. The generalized linear mixed model with binomial distribution was used for the analysis of the blood meal inhibition test data. The variables concentrations and cups represented the fixed and random effects respectively. The analysis required the lme4 package^52^.

### Ethics approval and consent to participate

The plant material in this study was used in accordance with the relevant regulations and recommendations.

## Results

### Identification of EO compounds

**Figure 3:**
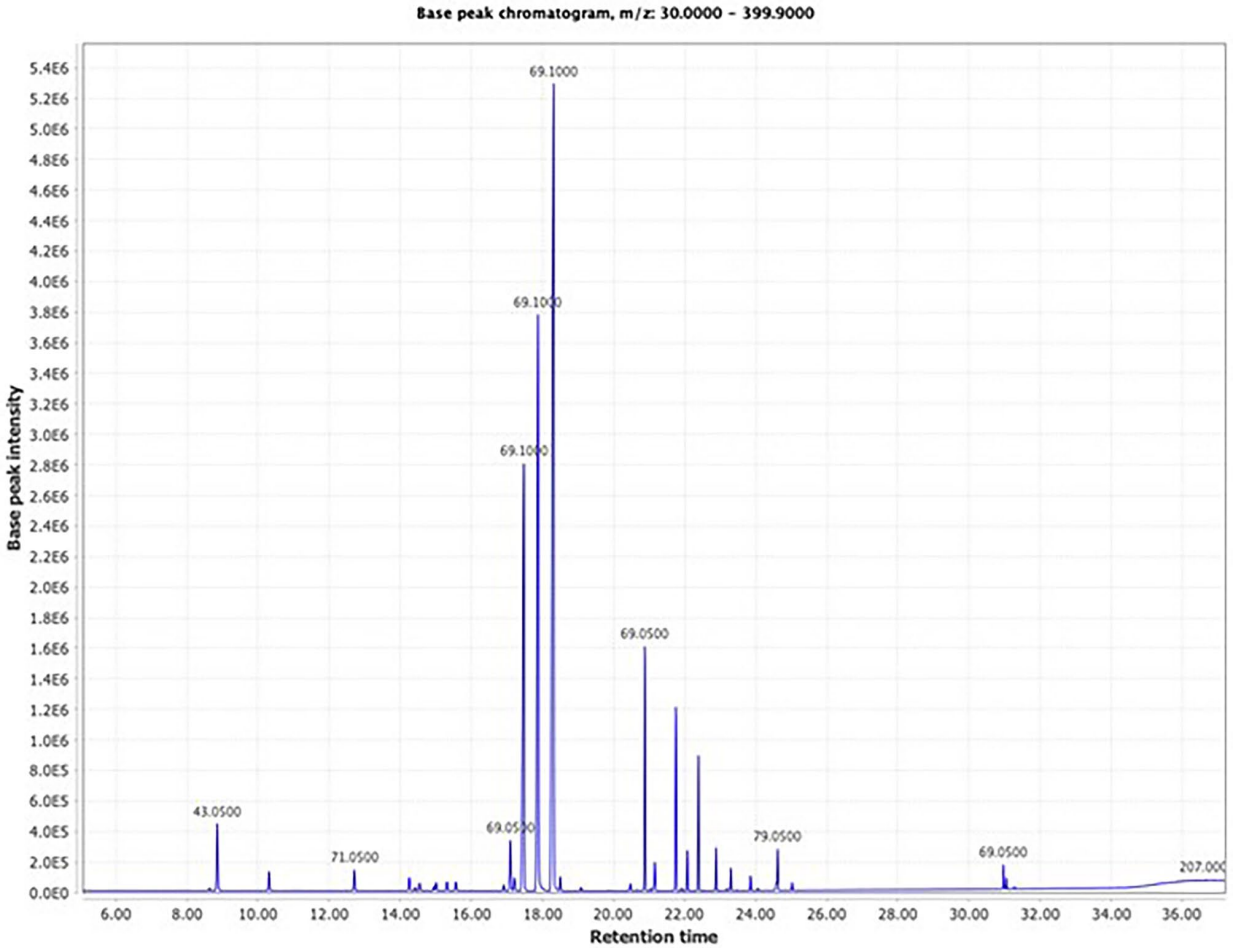
Total ion chromatogram of the essential oil extracted from *Lippia alba* leaves harvested in Abidjan (Côte d’Ivoire).

The analyses identified 24 compounds, the main ones being geranial (26.79%), neral (19.07%) and geraniol (14.52%) (Fig. 3, Table 1).

**Table 1:** Phytochemical composition of the essential oil of *L. alba* leaves harvested in Abidjan (Côte d’Ivoire).

Compounds	Rt (min)	Retention Indices (FAME)	Area (%)
6-methyl-5-hepten-2-one	8.845917	982.8687	2.05
Limonene	10.3026	1027.432	0.74
Linalool	12.70438	1097.762	0.87
Carane	14.41685	1146.995	0.18
Citronellal	14.54258	1150.607	0.41
z-isocitral	14.94143	1162.064	0.47
2-Butyrylfuran	15.00647	1163.932	0.13
Trans-limonene-1,2-oxide	15.56572	1179.996	0.67
Citronellol	16.90968	1223.006	0.30
Neral	17.46895	1242.874	19.07
Geraniol	17.87213	1257.197	14.52
Geranial	18.30567	1272.598	26.79
Neryl acetate	20.47335	1364.547	0.26
Lavandulyl acetate	20.87655	1383.185	4.28
α-ylangene	21.15835	1396.212	1.72
6,9-guaiadiene	22.0731	1445.048	2.20
Cis-ethyl-linalyl acetate	22.38525	1461.933	2.62
Longifolene	22.87948	1488.667	1.32
Alpha-cedrene	23.29568	1512.689	0.91
α-bulnesene	23.30002	1512.955	0.90
Caryophyllene	23.8506	1546.758	0.37
Epoxyvulgarone A	24.61363	1593.605	3.64
Citronellyl butyrate	25.02115	1620.407	0.54
(Z)-9-Hexadecenal	30.96493	2046.739	0.37

Rt, retention time.

### Adulticidal tests

The 0.1% EO produced very low KD60 and mortality *An. gambiae* individuals (7.8% and 4.5% respectively). The mortality recorded at this concentration was not significantly different from the negative control. From the 1% concentration onwards the KD60 and mortality rates were 100% except for the 2.5% which resulted in 97.6% mortality. Permethrin caused 100% KD60 and mortality at 0.011 and 0.11 mg/cm^2^ (Table 2).

**Table 2:** Mortality and KD60 (Knockdown at 60 min) of *L. alba* essential oil (EO) and permethrin against *An. gambiae* and *Ae. aegypti.*

			*An*. *gambiae* Kisumu			*Ae*. *aegypti* SBE		
			n	% KD60 (95% CI)	% Mortality (95% CI)	n	% KD60 (95% CI)	% Mortality (95% CI)
EO	0.1%	Control	86	0	10.4 (4.0–16.4)	82	0	0
		Treated	90	7.8* (2.2–13.2)	4.5^a^ (0–12.5)	84	3.6 (0–7.4)	2.3 (0–5.5)
	1%	Control	86	0	9.3 (3.2–15.4)	81	0	0
		Treated	88	100***	100^a^***	85	100***	16.4*** (8.6–24.2)
	2.5%	Control	77	3.89 (0–8.0)	1.2 (0–3.6)	80	1.16 (0–3.5)	1.1 (0–3.5)
		Treated	85	100***	97.6*** (94.4–100)	84	100***	64.2*** (54.0–74.4)
	5%	Control	87	0	13.7 (6.5–20.9)	80	5 (0.3–9.7)	2.5 (0–5.9)
		Treated	79	100***	100^a^***	83	100***	100***
Permethrin	0.011 mg/cm^2^	Control	87	0	5.7 (1.0–10.4)	83	0	1.2 (0–3.5)
		Treated	84	100***	100^a^***	80	100***	100***
	0.11 mg/cm^2^	Control	87	0	10.3 (4.0–16.6)	81	0	2.4 (0–5.7)
		Treated	83	100***	100^a^***	83	100***	100***

SBE, sensitive strain of *Ae. aegypti* from Benin; n, number of mosquitoes exposed, 95% CI, 95% confidence interval; ^a^, corrected mortality. The proportions of KD60 or dead mosquitoes induced by the essential oil were compared to those induced by the different negative controls using Fisher exact test; ***: P < 0.0001, **: P < 0.001, *: P < 0.05.

Contrary to the other concentrations, the KD60 and mortality induced by 0.1% EO on *Ae. aegypti* were not significantly different from the negative control. At 1%, KD60 was 100% but mortality was only 16.4%. The KD60 and mortality rates recorded with the 2.5% solution were 100% and 64.2% respectively. The 5% dose induced 100% KD60 and mortality. Permethrin caused 100% KD60 and mortality at 0.011 and 0.11 mg/cm^2^ (Table 2).

### Contact irritancy tests

The proportions of *An. gambiae* females escaping from the EO-treated tubes to the untreated tubes were 60.8% and 100% at the 0.1 and 1% doses respectively. These proportions decreased to 79.6% for the 2.5% concentration and 70.4% for the 5% concentration. At concentrations of 0.011 and 0.11 mg/cm^2^, permethrin caused 23.1 and 48.5% displacement of mosquitoes to untreated tubes, respectively (Table 3).

**Table 3:** Contact irritancy and repellent effects of *L. alba* essential oil (EO) on *An. gambiae* and *Ae. aegypti* (permethrin = positive control of contact irritancy tests).

			Contact irritancy				Non-contact repellency			
			*An. gambiae* Kisumu		*Ae. aegypti* SBE		*An. gambiae* Kisumu		*Ae. aegypti* SBE	
			n	% escaped (95% CI)	n	% escaped (95% CI)	n	% escaped (95% CI)	n	% escaped (95% CI)
EO	0.1%	Control	71	23.9 (14–33.8)	68	17.6 (8.6–26.6)	66	16.6 (7.7–25.5)	67	1.4 (0–4.2)
		Treated	69	60.8*** (49.3–72.3)	72	62.5*** (51.4–73.6)	66	68.1*** (56.9–79.3)	67	7.4 (1.2–13.6)
	1%	Control	67	17.9 (8.8–27)	69	14.4 (6.2–22.6)	71	18.3 (9.4–27.2)	71	7 (1.1–12.9)
		Treated	67	100***	71	91.5*** (85.1–97.9)	69	76.8*** (66.9–86.7)	70	68.5*** (57.2–79.8)
	2.5%	Control	70	24.2 (14.2–34.2)	67	7.4 (1.2–13.6)	66	4.54 (0–9.5)	63	1.5 (0–4.5)
		Treated	64	79.6*** (69.8–89.4)	68	94.1*** (88.5–99.7)	62	43.5*** (31.2–55.8)	68	33.8*** (22.6–45)
	5%	Control	69	17.3 (8.4–26.2)	65	4.6 (0–9.6)	63	9.5 (2.3–16.7)	64	1.5 (0–4.4)
		Treated	71	70.4*** (59.8–81)	71	77.4*** (67.7–87.1)	68	26.4* (16–36.8)	67	11.9* (4.2–19.6)
Permethrin	0.011 µl/cm^2^	Control	68	7.3 (1.2–13.4)	67	10.44 (3.1–17.7)	–			
		Treated	69	23.1* (13.2–33)	72	31.9* (21.2–42.6)				
	0.11 µl/cm^2^	Control	67	10.4 (3.1–17.7)	66	10.6 (3.2–18)				
		Treated	68	48.5*** (36.7–60.3)	70	60*** (48.6–71.4)				

SBE, sensitive strain of *Ae. aegypti* from Benin; n, number of mosquitoes exposed, 95% CI: 95% confidence interval. The proportions of displaced mosquitoes induced by the essential oil were compared to those induced by the different negative controls using Fisher exact test; *** :P < 0.0001, **: P < 0.001, *: P < 0.05.

The 0.1, 1 and 2.5% EO solutions recorded 62.5, 91.5 and 94.1% of *Ae. aegypti* mosquitoes escaping, respectively. At 5%, the proportion of escaped mosquitoes was 77.4%. Permethrin caused 31.9% displacement of mosquitoes at 0.011 mg/cm^2^ and 60% displacement at 0.11 mg/cm^2^ (Table 3).

### Non-contact repellency tests

With *An. gambiae*, the 0.1 and 1% EO solutions repelled 68.1% and 76.8% of mosquitoes respectively, while the 2.5 and 5% solutions produced 43.5% and 26.4% repellency respectively (Table 3).

At 1% EO, the proportion of escaped females was 68.5% for *Ae. aegypti* mosquitoes. Repellency rates were 33.8% for the 2.5% solution and 11.9% for the 5% solution (Table 3).

### Blood meal inhibition

**Figure 4:**
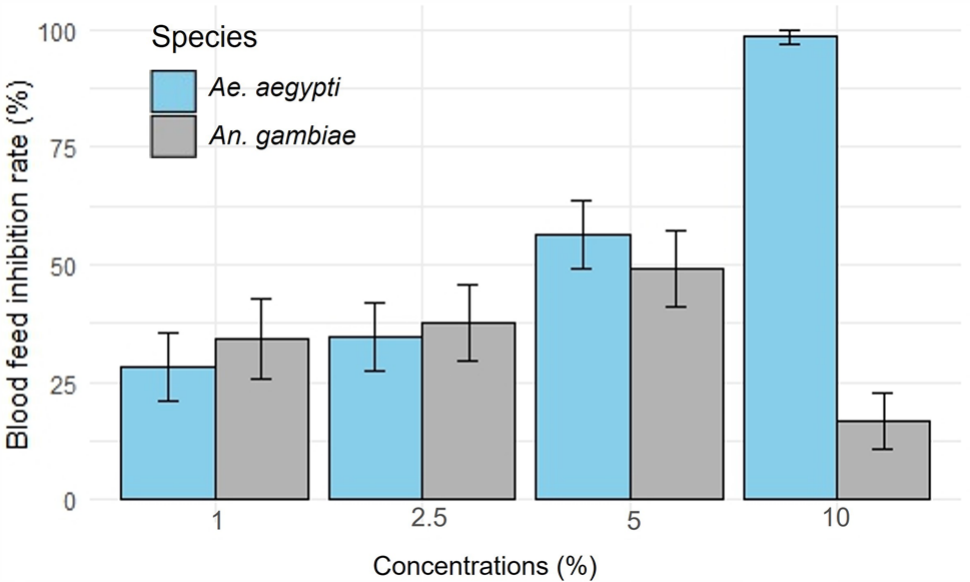
Blood feeding inhibition of *Lippia alba* essential oil against *An. gambiae* and *Ae*. *aegypti.*

The proportions of blood fed females did not significantly decrease with increasing concentrations in *An. gambiae* (Tables 4). The EO produced 34.2%, 37.5% and 49% blood feeding inhibition in *An. gambiae* at concentrations of 1%, 2.5% and 5% respectively. Surprisingly, a significantly lower inhibition (16.7%) was observed with the 10% concentration (Fig. 4).

**Table 4:** Feeding rate of *An. gambiae* and *Ae*. *aegypti* females exposed to membranes impregnated with essential oil of *L. alba*.

		*An. gambiae* Kisumu					*Ae. aegypti* SBE				
		n	% Feed (95% CI)	GLMM analysis			n	% Feed (95% CI)	GLMM analysis		
				Parameters	Intercept	Concentr.			Parameters	Intercept	Concentr.
1st phase	Control	205	80 (74.6–85.4)	Estimate	0.73	− 0.03	205	88.2 (83.8–92.6)	Estimate	1.46	− 0.29
	1%	201	52.7 (45.8–59.6)				215	63.2 (56.8–69.6)			
				SE	0.18	0.03			SE	0.23	0.05
	2.5%	224	50 (43.5–56.5)				223	57.8 (51.4–64.2)			
	5%	218	40.8 (34.3–47.3)	t-value	3.92	− 0.87	218	38.5 (32.1–44.9)	t-value	6.18	− 5.79
2nd Phase	Control	210	91.9 (88.3–95.5)				204	93.1 (89.7–96.5)			
				P	0.0003	0.3861			P	0	0
	10%	218	76.6 (71–82.2)				209	1.4 (0–2.9)			

SBE, sensitive strain of *Ae. aegypti* from Benin; n, number of mosquitoes exposed; 95% CI, 95% confidence interval; GLMM, Generalized Linear Mixed Model; Concentr., concentrations; number of observation: 54; number of mosquito cups per specie: 54; SE, standard error of parameter estimate; t-value, estimate to standard error ratio; P, statistic for t-value.

The mean rates of blood fed *Ae. aegypti* significantly decreased as concentrations increase (Table 4). The blood feeding inhibition rates increases from 28.4% for the 1% concentration to 98.5% for the 10% concentration (Fig. 4).

## Discussion

The management of mosquito resistance to chemical insecticides and the biting behaviour of some species are motivating the search for complementary and/or alternative control methods. We identified the molecules present in the essential oil of *L. alba* and determined its biological effects (insecticidal, repellent and blood meal inhibition) on *An. gambiae* and *Ae. aegypti*. Mainly composed of citral, the EO has been shown to be toxic, irritant and repellent against both species of mosquito. Unexpectedly, the lowest blood meal inhibition rate was recorded with the highest dose of EO with *An. gambiae*, while this was not observed with *Ae. aegypti.* These results provide the basis for future work on the impact of *L. alba* EO formulations for protection against mosquito bites in the field.

The EO evaluated in this study consists mainly of citral (45.86%, geranial and neral being the two isomers of citral) and geraniol (14%). The chemotypes of *L. alba* EOs, which can be associated with the genetic forms of the plant species, have been characterised according to their main compounds, the variability of which can be related to the morphological characteristics of the leaves and the localities or environmental conditions of production^29,53–59^. Hennebelle et al.^53^ classified the EOs of *L. alba* into seven chemotypes on the basis of the phytochemical profiles of 4 samples from the French overseas departments and those of 54 other samples reported in 35 publications. This classification can be summarised as follows: (I) citral, linalool, caryophyllene as the major compounds (four subtypes); (II) tagetenone; (III) limonene, carvone in variable amounts, often replaced by related biosynthetic monoterpene ketones (two subtypes); (IV) myrcene; (V) γ-terpinene; (VI) camphor-1,8-cineole and (VII) estragole^29^.

The insecticidal activity of some EOs may be related to the inhibition of mitochondrial enzymes and acetylcholinesterase (AChE) activities by their compounds^60,61^. In this study, the EO of *L. alba* has been shown to be toxic to insecticide susceptible strains of mosquitoes, as it induced 100% KD60 in *An. gambiae* and *Ae. aegypti* at doses as low as 1%. Dua et al.^18^ also obtained 100% KD60 with the EO of *L. camara* another Verbenaceae mainly composed of caryophyllene, eucalyptol, *α*-humelene and germacrene-D, at a dose of 4% with *Anopheles culicifacies*, *Anopheles fluviatilis*, *Anopheles stephensi*, *Cx. quinquefasciatus* and *Ae. aegypti*. The carvone chemotype of *L. alba* EO at 0.1% induced 80% mortality in *Ae*. *aegypti* adults^39^, whereas the citral chemotype evaluated in the present study induced 4.5% mortality in *An. gambiae* and 2.3% in *Ae. aegypti* at the same dose. This divergence in mortality can be explained either by the higher insecticidal effect of carvone chemotype or by the use of the CDC bottle by Castillo et al.^39^ and the fact that EO deposited on glass may be more effective than on paper that could adsorb a part of the EO. Significant mortalities were observed in this study from 1% with *An. gambiae* and 2.5% with *Ae. aegypti*. Larvicidal activity of several chemotypes of *L. alba* EO against *Ae. aegypti*, *Ae. albopictus* and *Cx. quinquefasciatus* has also been reported^40,62–64^. Moreover, the carvone and citral chemotypes of *L. alba* have been shown to be toxic against other insects (*Ulomoides dermestoides, Sitophilus zeamais and Tribolium castaneum*)^65,66^ and *Rhipicephalus* (*Boophilus*) *microplus* tick^67,68^.

The 1% solution of *L. alba* EO gave 100% KD60 but only 16.4% mortality with *Ae*. *aegypti*. These results have also been observed when using intermediate concentrations of EOs as for instance 3–5% solutions of *C. nardus, N. cataria* and *O. americanum* which caused 100% KD60 and less than 10% mortality on *Ae. aegypti*^69^. The recovery of *Ae*. *aegypti* females after knockdown may be explained by natural detoxification mechanisms or by reversible inhibition of receptors in the nervous system as observed with some insecticides, including natural pyrethrins^70,71^.

In the present study, the 1% concentration induced 76.8 and 68.5% repellency on *An*. *gambiae* and *Ae. aegypti* respectively. The 2.5 and 5% concentrations gave lower repellent rates than those obtained with 1% on both species. As the tests were carried out in hermetically closed cylinders, the 2.5 and 5% concentrations could have started to intoxicate the mosquitoes or impaired their sensory systems. Cutaneous application of the citral chemotype of *L. alba* EO protects against mosquito bites^41^. The EO of *L. alba*, composed mainly of 2, 7 octadione-1-butoxy and 2-isopropenyl-5-methyl hex-4-enol, has a repellent activity, unlike that composed mainly of carvone and limonene^39,40^. Odour Binding Proteins (OBPs) transport volatile molecules to olfactory receptors which reside in contact with the dendrites of sensory neurons. Citral and limonene showed good docking with OBP_1_ and OBP_22_, which have been identified as mediators of olfaction in *Ae. aegypti*, in addition to acetylcholinesterase^72,73^. One of the potential mechanisms of action of the repellent compounds in EOs such as *Lippia thymoides*, *Cymbopogon winterianus*, *Eucalyptus globulus* and *L. alba* is their interaction with OBP_1_^41^.

The irritant effect observed in the contact repellency test may not only be as a consequence of the intrinsic irritancy of the EO, since a spatial repellency effect was observed for the two mosquito species. In fact, the movement of mosquitoes away from a treated surface without and after contact involves the binding of molecules to specific olfactory receptors on the antennae and specific gustatory receptors on the tarsi, respectively^74^. These two mechanisms would therefore have resulted in the escape of *An. gambiae* and *Ae. aegypti* females from tubes treated with *L. alba* EO to untreated tubes during contact irritancy tests. This irritancy increased from 60.8 to 100% from 0.1 to 1% concentrations, but fell to 79.6% and 70.4% respectively at 2.5% and 5% on *An. gambiae*. In *Ae. aegypti*, the proportion of escaped females was 77.4% with the 5% solution, compared to 94.1% with 2.5%. As the EO showed a neurotoxic effect, the apparent reduction of irritancy are likely to be due to some mosquitoes starting to be intoxicated or being knocked down during the 10 min the slide unit was opened. Reduced irritability caused by a knock down effect has already been observed with DEET and IR3535 against *Ae. aegypti*^75^.

The proportions of blood fed *An. gambiae* ranged from 34 to 49% with 1–5% concentrations of *L. alba* EO. Wangrawa et al.^76^ obtained lower proportions of blood fed females using tunnel tests: 20.5% with 1.5% *L. cam*ara, 30.3% with 3% *Ocimum canum*, 35.3% with 2.5% *Hyptis spicigera* and 19.5% with 2% *Hyptis suaveolens*. This may be due to the fact that tunnel tests not only measure the direct blood feeding inhibition but also the combined effects of mortality and excito-repellency when mosquitoes are exposed to a treated netting. The highest rate of blood fed females (76.6%) was observed with the 10% concentration of *L. alba* EO on *An. gambiae* in the present study. This unexpected observation has already been reported by Hodjati and Curtis^77^, who obtained higher feeding rates with the highest dose of permethrin on susceptible and resistant populations of *An. stephensi*. This high dose was also associated with the lowest KD and mortality in their study. *L. alba* EO had a better effect on blood meal inhibition of *Ae. aegypti* with an inhibition rate of 98.4% for the 10% concentration. The behavioural differences found in both species as well as the unexpectedly high blood feeding rate of *An. gambiae* at high concentration will necessitate further investigations.

## Conclusion

The investigations carried out showed that the EO of *L. alba* had very promising knockdown, toxicity, irritant and repellent effects as well as a blood feeding inhibition on *An*. *gambiae* and *Ae. aegyti*. It is clearly a good candidate for the development of sustainable biological products for mosquito control. To this end, further evaluations under field conditions should be carried out to measure the effect of the extract on mosquito bites and/or pathogen transmission. The results obtained have prompted also an evaluation of the biological effects of EOs of *L. alba* harvested in different localities of west Africa against mosquito vectors of pathogens. Finally, it will be interesting to investigate the impact high doses of *L. alba* EO on odorant receptors of *An. gambiae* and their consequences on mosquito behaviour.

## Data Availability

The datasets generated during and/or analysed during the current study are available from the corresponding author on reasonable request.
